# Unraveling the COVID-19 hospitalization dynamics in Spain using Bayesian inference

**DOI:** 10.1186/s12874-023-01842-7

**Published:** 2023-01-25

**Authors:** Alberto Aleta, Juan Luis Blas-Laína, Gabriel Tirado Anglés, Yamir Moreno

**Affiliations:** 1 ISI Foundation, Via Chisola 5, 10126 Torino, Italy; 2 Institute for Biocomputation and Physics of Complex Systems (BIFI), University of Zaragoza, 50018 Zaragoza, Spain; 3 Servicio de Cirugía General y Aparato Digestivo (Jefe de Servicio), Hospital Royo Villanova, Av San Gregorio s/n, 50015 Zaragoza, Spain; 4 Unidad de Cuidados Intensivos (Jefe de Servicio), Hospital Royo Villanova, Av San Gregorio s/n, 50015 Zaragoza, Spain; 5 Department of Theoretical Physics, University of Zaragoza, 50018 Zaragoza, Spain; 6 Centai Institute, 10138 Torino, Italy; 7 Complexity Science Hub, 1080 Vienna, Austria

**Keywords:** Bayesian inference, Hospitalization dynamics, Covid-19, Regional differences, Public health

## Abstract

**Background:**

One of the main challenges of the COVID-19 pandemic is to make sense of available, but often heterogeneous and noisy data. This contribution presents a data-driven methodology that allows exploring the hospitalization dynamics of COVID-19, exemplified with a study of 17 autonomous regions in Spain from summer 2020 to summer 2021.

**Methods:**

We use data on new daily cases and hospitalizations reported by the Spanish Ministry of Health to implement a Bayesian inference method that allows making short-term predictions of bed occupancy of COVID-19 patients in each of the autonomous regions of the country.

**Results:**

We show how to use the temporal series for the number of daily admissions and discharges from hospital to reproduce the hospitalization dynamics of COVID-19 patients. For the case-study of the region of Aragon, we estimate that the probability of being admitted to hospital care upon infection is 0.090 [0.086-0.094], (95% C.I.), with the distribution governing hospital admission yielding a median interval of 3.5 days and an IQR of 7 days. Likewise, the distribution on the length of stay produces estimates of 12 days for the median and 10 days for the IQR. A comparison between model parameters for the regions analyzed allows to detect differences and changes in policies of the health authorities.

**Conclusions:**

We observe important regional differences, signaling that to properly compare very different populations, it is paramount to acknowledge all the diversity in terms of culture, socio-economic status, and resource availability. To better understand the impact of this pandemic, much more data, disaggregated and properly annotated, should be made available.

**Supplementary Information:**

The online version contains supplementary material available at 10.1186/s12874-023-01842-7.

## Background

During the early stages of the COVID-19 pandemic, one of the greatest public health concerns was the adequacy of healthcare resources to treat infected cases. With cases growing exponentially, and roughly 10% of the detected cases needing hospitalization in intensive care units, it was soon realized that national health systems could be easily overwhelmed [1–3]. To slow the advance of the epidemic and protect national health systems, many countries implemented strict lockdowns during the first wave of the epidemic. Further waves also forced the application of important non-pharmaceutical interventions, reducing the effective reproduction rate of the disease and preventing the collapse of the healthcare systems [4–7].

At the same time, this event is the largest pandemic in the Digital Age, with wide access to the internet and ubiquity of social networks, representing a complete paradigm shift in terms of communication, data collection, storage, and dissemination at all scales [8, 9]. Yet, many healthcare systems were ill-prepared for an event of such scale. For instance, a group of researchers, journalists and non-profit organizations studied the COVID-19 data dashboards for each state in the US and concluded that the US lacked “standards for state-, county-, and city- level public reporting of this life-and-death information”, calling it an “information catastrophe” [10]. Many numbers had important lags, with a quarter of deaths reported less than 6 days after they had occurred while another 25% were reported more than 45 days later [11]. Furthermore, even if open data was available, it often lacked the granularity necessary to understand the gender, racial, ethnic and economic disparities created or amplified by the pandemic [12].

Similar problems can also be found in many countries in the EU [13]. In the case of Spain, during the first wave, the reporting delay was highly variable, ranging from a few days in early March to over 2 weeks in April [14]. The problem was further exacerbated due to the high decentralization of the health system in Spain, managed by each autonomous region independently, which caused many problems of data synchronization and case definition [15]. In March 2021, already 1 year into the pandemic, a report on child mortality of COVID-19 in Europe showed that Spain had the highest mortality of all the countries analyzed [16]. A deeper investigation revealed that some patients over 100 years old had been incorrectly labeled as underaged, resulting in an abnormal number of children deaths which was later amended, yielding results compatible with other European countries [17]. Thus, it is important to devise methodologies that can fully exploit these public data sources and detect errors, inconsistencies or underlying methodological changes that are not correctly addressed by the providers.

Besides these considerations regarding the use of data, and despite its importance, the literature on short-term predictions of bed occupancy for the COVID-19 pandemic is relatively limited [18]. The earliest models were adapted from the Erlang loss models used to dimension isolated wards [19, 20], but their main limitation was that they assumed a constant influx of patients, something unsuitable for the dynamics of the COVID-19 pandemic [21]. To solve this, Baas et al. used a network of infinite server queues to predict the occupancy in individual hospitals. However, their method focuses on predicting occupancy at single hospitals for which they require a complete record of time stamps for patient admissions, transfers and discharges [3]. Bekker et al. follow a quite different approach since they try to avoid the use of any external information [18]. Their model is based on previous admissions, together with information on individual length of stay (LoS) and can provide accurate 7-day ahead predictions. They also provide a good overview of the related literature for occupancy predictions both for COVID-19 and other scenarios [18]. Castro et al. followed a similar approach given the lack of consistent and timely hospitalization data [22].

Other authors explored classical disease transmission models. These modeling works tend to study the whole dynamics of the pandemic and the effect of non-pharmaceutical interventions, assuming a certain rate of hospitalization while focusing on infections and deaths [5, 7, 23–26]. Along these lines, Campillo-Funollet et al. used a SEIR-D model with 8 parameters to estimate both the transmission and hospitalization rates under the assumption of no policy changes within the period under study [27]. Chin et al. analyzed the forecasting power of some influential models and found them to be “highly inaccurate” for ICU bed utilization [28]. Donker et al. based their predictions on the value of another important parameter of epidemic models, the reproduction number (Rt), but failed to account for the introduction of non-pharmaceutical interventions, leading to an important overestimation of bed occupancy [29]. Garcia-Vicuña et al. used general population growth models to predict new admissions [30]. Lastly, de Barros Braga et al. proposed an approach based on artificial neural networks which provided better results for cumulative variables than for daily estimations [31].

A crucial element on any of these models is to account for the regional differences, something that many papers - especially those focused on modeling - seldom do. Indeed, both the admission rate and the length of stay may depend on the specific characteristics of the patients, the socioeconomic status of the population, or by local or national policies. For instance, in two populations with the same socioeconomic characteristics and transmission dynamics, the admission rate will be lower in the one with the largest testing capacities. Similarly, the LoS might be shorter in areas with low hospitalization capacity. Further, these differences may evolve in time following policy changes. As such, it is also important to contextualize properly the period under study.

In this context, Hoekman et al. studied the number of COVID-19 hospitalized patients per 100,000 inhabitants in the Netherlands at the regional level, to avoid testing bias, during the first wave [32]. They found large heterogeneity with higher hospitalization rates in the initial epicenter. Similar results for the first wave were found in Italy [33], Saudi Arabia [34], Scotland [35] or New York City [36]. In terms of LoS, an early systematic review conducted in April 2020 found a median of 14 days in China and 5 days in the rest of the world [37]. Using individual patient records of the first wave, Vekaria et al. estimate the average LoS to be between 8 and 9 days in the United Kingdom [38]. Similarly, López-Cheda et al. used individual records from patients in the region of Galicia, Spain, and obtained a median LoS of 11 days. However little work exists in relation to regional differences in hospital dynamics, and even less after the first wave.

In this paper, we focus on the context of hospital dynamics and present a simple model to translate detected incidence into hospital admissions and bed occupancy. Initially, the model was implemented to estimate bed occupancy in the autonomous region of Aragon during the fourth wave of the pandemic, but it is here applied to study the temporal dynamics of hospitalizations of other regions of the country too. Besides, we also show that this tool can also be used to detect changes underlying the data which cannot be easily detected otherwise. Lastly, we apply the methodology to compare the evolution of the 17 autonomous regions in Spain and relate it to the testing policy implemented in each region. Our methodology can therefore be used to scrutinize the unfolding of the disease and its impact, together with the NPIs adopted, on hospital occupancy. As a result, it can also be employed as a tool for preparedness and healthcare planning, alleviating the high and yet-to-assess delays in diagnosis and treatment of other -potentially deadly, e.g., cancer, tuberculosis, etc.- diseases caused by the pandemic.

## Methods

### Overview

The model estimates daily admissions and bed occupancy based on the daily incidence data reported by the authorities. To do so, we estimate the probability of being admitted into a hospital upon being confirmed as a positive case, as well as the delay between detection and admission, and the length of stay (LoS). We employ a Bayesian inference model trained on data from July 2020 to late November 2020, inspired by [4,47]. This technique allows us to integrate prior information with data and is flexible enough as to be easily applied to other regions. With this information, we can reproduce the hospital dynamics in the autonomous region of Aragon -taken here as a case study- from December 2020 to June 2021, and produce forecasts on hospitalization based on any technique for predicting the daily incidence. In addition, we show the generality of the methodology by applying it to all the autonomous regions of Spain. This allows us to study their evolution, and analyze their differences, strategies for case detection and the effects of the first stages of the vaccination campaigns.

### Datasets

We use the open data provided by the Spanish Health Ministry, which reports the number of confirmed cases by symptom onset, or notification date minus 3 days if it is not available, and the number of daily hospital admissions [39]. We further complement this dataset with the one provided by the regions of Aragon and Catalonia for some specific analysis [40, 41]. In particular, the main dataset provided by the Spanish Health Ministry does not contain information on discharges or bed occupancy, limiting regional comparisons to new admissions.

There is an alternative repository focused on hospitalization but has several gaps since it is not updated on Saturdays, Sundays and special holidays [42]. Furthermore, the data on discharges “might not be collected exhaustively.” Thus, for the analysis involving bed occupancy, we resort to the regional repository. Note also that the quality, accessibility and depth of each regional repository varies greatly, and not always matches the data provided by the Ministry, as we show later for the case of Catalonia. Another example is the incidence data provided by the government of Aragon, which is based on the notification date, while the incidence data provided by the Spanish Health Ministry is based on symptoms’ onset. Therefore, we perform the comparisons among different regions using the standardized data provided by the Spanish Health Ministry.

### Model

For each region *r*, given the number of daily cases on day *i*, ${C}_i^r$, we estimate the daily number of hospital admissions at time *t* as

1 $${A}^r(t)=\sum\limits_{i=0}^{t-1}{C}_i^r{p}_H^r{\upphi}^r\left(t-i\right)$$

where ${p}_H^r$ is the regional probability of hospitalization and *ϕ*^*r*^(*t* − *i*) is a delay function that yields the probability of being admitted to the hospital on day *t* given that the case was notified on day *i*. Data on the characteristics of all hospitalized individuals between early August and late November provided by the Health Department of the region of Aragon indicates that *ϕ* can be approximated by a Half-Cauchy distribution:

2 $$f\left(x|\beta \right)=\frac{2}{\pi \beta \left[1+{\left(\frac{x}{\beta}\right)}^2\right]}$$

where β is the scale parameter. Similarly, to determine the daily number of discharges, we can apply a convolution between the number of daily admissions and the distribution of the length of stay, ψ,

3 $${\displaystyle \begin{array}{c}{D}^r(t)=\sum\limits_{i=0}^{t-1}{A}^r(i){\uppsi}^r\left(t-i\right)\end{array}}$$

In this case, the data showed that ψ can be well approximated by a log-normal distribution:

4 $${\displaystyle \begin{array}{c}f\left(x|\upmu, \upsigma \right)=\frac{1}{x\sqrt{2\uppi \upsigma}}\mathit{\exp}\left(-\frac{{\left(\mathit{\log}x-\upmu \right)}^2}{2\upsigma}\right)\end{array}}$$

where μ is the location parameter and σ the standard deviation. So that the bed occupancy in region *r*, on day *t*, given the daily incidence up to that day will be given by

5 $${\displaystyle \begin{array}{c}{B}^r(t)=\sum\limits_{i=0}^t\left({A}^r(i)-{D}^r(i)\right)\end{array}}$$

In the Supplementary Information (Figs. S8-S10), we also explore the results when the admission delay is approximated by an Exponential distribution and the length of stay by a Gamma distribution.

### Bayesian inference

To estimate the parameters, we implement a Bayesian statistical model using PyMC3 3.11.5 with Python 3.8 [43]. Note that all parameters cannot be estimated simply from bed occupancy data (Eq. (5)) since a larger probability of hospitalization with small LoS can yield the same occupancy as a small probability of hospitalization with large LoS. Thus, we first estimate the set of model parameters for the daily admissions, ${\uptheta}^r=\left\{{p}_H^r,{\upbeta}^r,{\alpha}^r\right\}$, using Bayesian inference with Markov-chain-Monte-Carlo (MCMC). We assume that the likelihood of observing the real-world data point, ${\hat{A}}^r(t)$, is given by NegBinomial(μ = *A*^*r*^ (*t*| θ), α) to allow for over-dispersion. We run 4 independent chains using NUTS with 2000 burn-in steps and 5000 steps to approximate the posterior distribution of the parameters with uninformed priors.

With these parameters and Eq. (1) we can obtain the daily number of admissions as a function of the number of new detected cases. This information, together with Eq. (5) and the observed bed occupancy, ${\hat{B}}^r(t)$, can be used to estimate the parameters of the log-normal distribution governing discharges. In this case we assume that the likelihood of observing the real data point is given by Normal(μ = *B*^*r*^(*t*| θ), σ), but otherwise the training procedure is the same as for the admissions.

### Forecasting

The previous model can translate new infections into new admissions, use the distribution of LoS and, finally, estimate bed occupancy for the short-term (2-3 days). Thus, to increase the prediction window, it is necessary to forecast future incidence. There are many techniques available to do this, ranging from classical compartmental models to Bayesian inference akin to the one used here, detailed agent-based models, metapopulation models, branching processes, machine learning and deep learning techniques or other time-series forecasting tools [44–50]. Since this is not the main purpose of this work, we resort to a simple heuristic that yields satisfactory results and is easier to communicate to non-technical stakeholders.

First, we computed the evolution of the effective reproduction number in the region of Aragon from early July to mid-November. Then, we explored how this quantity changed from day to day by dividing the value at time *t*, *R*(*t*), over its value on the previous day, *R*(*t* − 1). This analysis revealed that during the growing phase of an outbreak, the daily increase on *R*(*t*) is below 5%. Similarly, during the shrinking phase induced by the non-pharmaceutical interventions imposed by the authorities, the daily decrease is close to 5%, see Fig. S3. Given this observation, we can forecast the value of the reproduction number as

6 $${\displaystyle \begin{array}{c}R\left(t+i\right)=\pm 1.05R\left(t+i-1\right)\end{array}}$$

with the sign chosen depending on whether the outbreak is growing or decreasing. Then, we can estimate the number of new cases by multiplying this quantity iteratively by the number of cases observed or estimated in the previous days and introduce it in Eq. (5) to forecast bed occupancy.

## Results

### Estimating model parameters for daily admissions

**Fig. 1:**
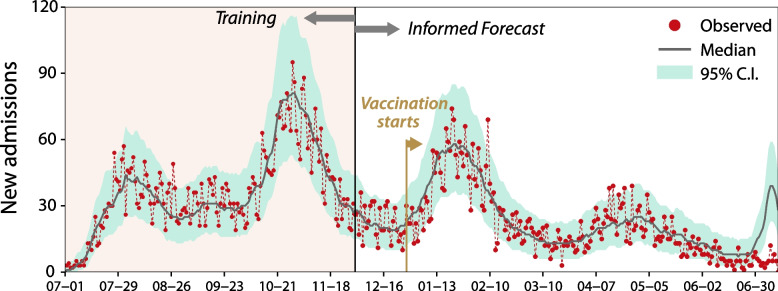
Number of daily admissions to hospitals in the region of Aragon. To estimate the parameters of the model, we use the information available up to December 1, 2020. From December 1 the number of admissions is obtained using the estimated parameters and the observed number of daily detected cases, applying Eq. (1). The solid line represents the median value of the estimation and the shaded area the 95% C.I, while the dots represent the observed data. Note that on December 28 the vaccine roll-out started in Spain. The estimated parameters are $\left\{{\textrm{p}}_{\textrm{H}}^{\textrm{AR}},{\upbeta}^{\textrm{AR}},{\upalpha}^{\textrm{AR}}\right\}={0.09, 3.56, 38.06}$

To estimate the model parameters, we choose the period between July 1, right before the second wave of infections started in Spain, and December 1, right before the beginning of the Christmas wave. In Fig. 1, we show the number of daily admissions obtained using Eq. (1), while in Fig. S1 we depict the corresponding prior and posterior distributions. The period before December 1 can be regarded as the training period for the model. Afterwards, the prediction is labeled “informed forecast” since it requires knowledge of the number of new cases.

We estimate an admission probability of ${p}_H^{\textrm{AR}}=$ 0.090 [0.086-0.094] (95% C.I.), while the scale parameter of the Half-Cauchy distribution governing hospital admission is β^AR^= 3.557[2.579-4.564] (95% C.I.). More specifically, this distribution yields a median interval of 3.5 days and an IQR of 7 days for the delay between case detection and hospital admission.

### Estimating model parameters for daily discharges

**Fig. 2:**
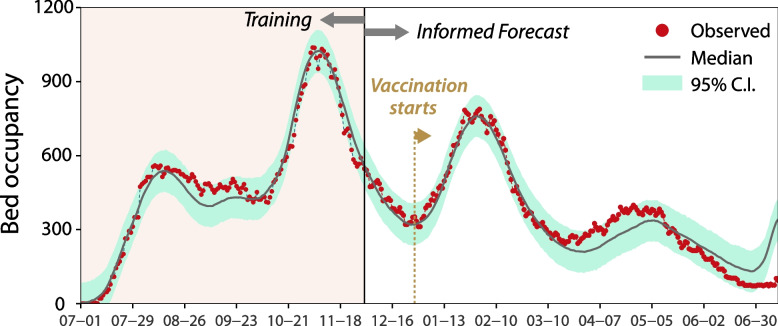
Daily number of beds occupied by COVID-19 patients in Aragon. To estimate the parameters of the model, we used information up to December 1, 2020. From December 1, the occupancy is obtained using the number of new detected cases, together with Eqs. (1) and (5). The solid line represents the median value of the estimation and the shaded area its 95% C.I., while the dots represent the observed data. Note that on December 28 vaccine roll-out started. The estimated parameters are $\left\{{\upmu}^{\textrm{AR}},{\upsigma}^{\textrm{AR}},{\upsigma}_{\textrm{N}}^{\textrm{AR}}\right\}={2.48, 0.62, 42.63}$

As described in the Methods section, we use the information on bed occupancy to estimate the model parameters for daily discharges. In Fig. 2, we show the observed daily occupancy of hospital beds in Aragon together with the estimated one. We observe that the agreement is exceptionally good, even after vaccination started, until the beginning of April 2021. From that point on, the estimated occupancy is lower than the observed one. Given that the estimated admissions still agree with the observed values of this variable, this implies that the length of stay for the age groups admitted to the hospital during those dates is larger than the average. Whether this is the effect of protecting the oldest individuals from severe infection, or due to the circulation of new variants, is something that cannot be addressed unless more disaggregated data is released (e.g., discharges by age-group).

We estimate that the Log-Normal distribution used to approximate the distribution on the LoS has μ^AR^= 2.476 [2.441-2.512] (95% C.I.) and σ^AR^= 0.620 [0.554-0.688] (see Fig. S2 and Table S1 for a summary of the estimated parameters). This distribution yields a median of 12 days with an IQR of 10 days.

### Bed occupancy in Aragon

**Fig. 3:**
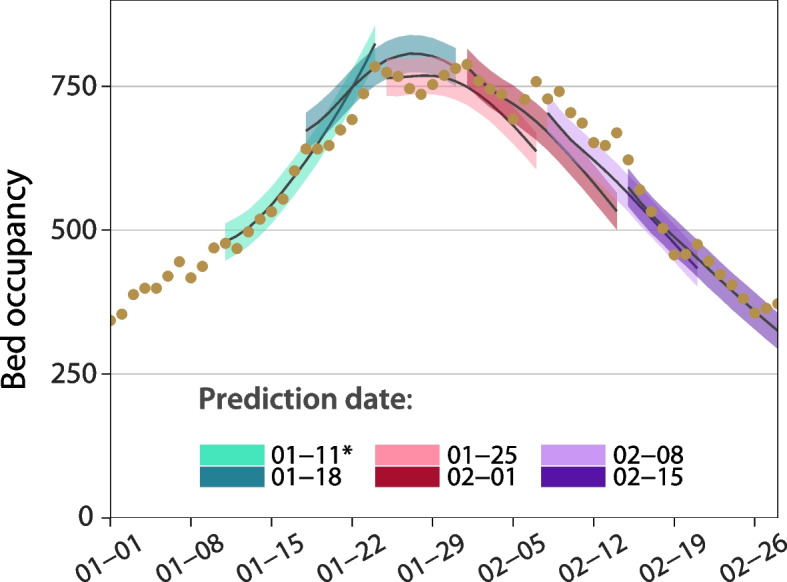
Forecasting bed occupancy in Aragon during the 2020-2021 Christmas wave. Dots show the actual value of bed occupancy, while solid lines represent the median value of the forecast, and their shaded regions display the 95% C.I. of the forecast for the week starting at the indicated date. At each date depicted in the figure, rather than using the observed incidence as in Fig. 2, the prediction algorithm runs on the number of cases detected up to that date and forecasts occupancy assuming a certain change of R(t). In the prediction made on January 11, 2021, the change is assumed to be + 0%. Following the introduction of several containment measures on January 15, the daily change was assumed to be − 5%

In Fig. 3, we show the forecasts performed during the Christmas wave. Each Monday, the algorithm ran on the data collected up to that date and forecasted bed occupancy as a function of the expected change of *R*(*t*). Three scenarios were considered: pessimistic (+ 5%), neutral (+ 0%) and optimistic (− 5%), which could be chosen depending on domain expertise and the unfolding of events, such as the imposition of new containment measures (see the section Surveillance data in Aragon in the Supplementary Material for the rationale behind this choice). A summary of all the estimated parameters used in the forecast is presented in Table S1.

### Estimation of new admissions in the rest of Spain

The model can be used to compare the characteristics and temporal evolution of the outbreaks in each autonomous region of Spain. This comparison is especially interesting since each region manages its own healthcare service and response to the pandemic, which might lead to different values for the parameters of the model. Thus, we have implemented the model to estimate new admissions for each of the 17 autonomous regions in Spain (the 2 autonomous cities, Ceuta and Melilla, have been discarded due to their small size). In Fig. S5, we show the number of new daily admissions up to May 1, 2021, in each region. As in the previous case, we compute their corresponding parameters $\left({p}_H^r,{\upbeta}^r\right)$ using data from July 1, 2020, to December 1, 2020. The model can estimate very well the number of new admissions in all regions, although there are some territories in which the estimation is slightly worse (see Table S2).

**Fig. 4:**
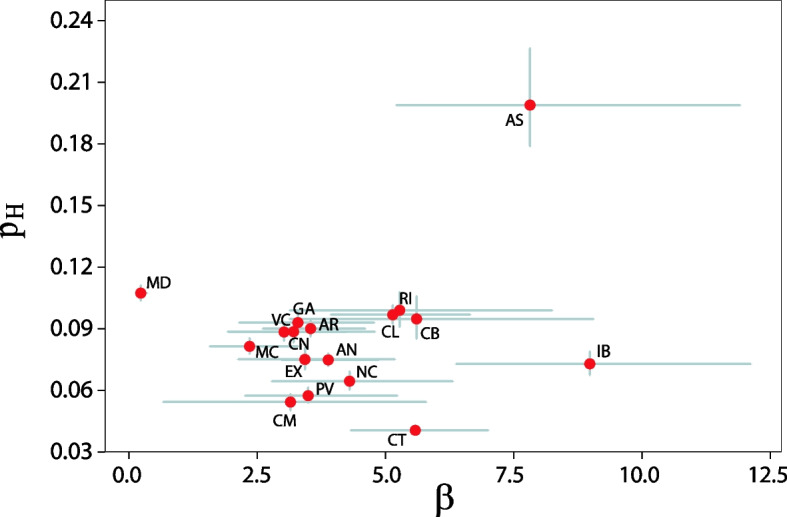
Admission dynamics in each region of Spain. Estimated value of the probability of being admitted into the hospital upon detection, p_H_, versus the parameters of the Half-Cauchy distribution governing the delay between detection and admission, β. The horizontal and vertical errorbars indicate the 95% C.I. Labels represent the ISO abbreviation of each autonomous region in Spain. The complete list of equivalences in shown in Table S2

In Fig. 4, we present a summary of the ${p}_H^r$ and β^*r*^ values obtained in the estimation of new admissions for all the autonomous regions of Spain. We observe that most regions cluster together around the same values, with a value for the probability of an infected individual needing hospital care upon detection around 9% and a delay of 3 to 4 days between detection and admission.

## Discussion

COVID-19, the largest pandemic of the Digital Age, has revolutionized the way in which public health information is created, stored and shared. At the same time, it has highlighted the weaknesses of the information systems of many governmental and healthcare institutions. Even when the data is openly shared, the lack of information on how it was obtained, continuous changes in government policies, and availability of resources, makes it challenging to use the data and perform proper comparisons between different regions. This can be easily seen when data from various sources, or from the same source but for different observables, are compared.

The model presented here focuses on incidence and bed occupancy data. In particular, it reproduces the dynamics associated with hospitalization as a function of the number of detected cases. In the case of Aragon, the hospital admission distribution yields a median interval of 3.5 days and an IQR of 7 days, which is remarkably close to the estimated values based on individual data provided by the regional government (median 4 days, IQR 6 days). Note that minor differences are expected since the way in which hospitals record case detection and admission might not be the same as the one provided by the Ministry of Health. For instance, while the Ministry of Health reports new cases as a function of symptoms’ onset, the regional government provides new cases based on notification date.

Similarly, we estimate that the distribution on the length of stay has a median of 12 days with an IQR of 10 days. Data provided by the regional government shows that the median value is between 8 and 9 days with an IQR of 8 days for surviving patients, while for deceased patients the median LoS is 9 days and the IQR is 11 days. Note that in our model we do not distinguish the reason for discharge. Besides, even though this quantity is less prone to biases due to the quality of the surveillance system, some noise might be introduced depending on the discharge policy (for instance, it is uncommon to discharge patients during weekends). Nevertheless, these values are compatible with those provided by the regional authorities, as well as the ones found in the literature for other parts of the world [37].

### Hospital admissions in Aragon

Focusing on the estimation of new hospital admissions in Aragon, a few remarks are in order. First, the region of Aragon was severely hit during the training period, with two infection waves, while other Spanish regions only had one. Second, the number of daily admissions can vary substantially, yielding a large prediction credible interval. Third, we observe that the prediction is reliable until late May since the probability of hospitalization estimated in summer 2020 still gives satisfactory results until that date. Lastly, the rising wave of infections in early summer 2021 was associated specifically with individuals between 15 and 25 y.o., and several outbreaks were linked to summer parties celebrated after the end of the academic year, leading to the closure of nightlife in several regions [51]. A similar pattern was also observed in the Netherlands [27]. This significant difference in the age profile explains why the incidence is not translated into hospital admissions.

There are several hypotheses that may explain why the probability of hospitalization barely changes even after the introduction of vaccination during the first half of 2021. First, early vaccination was focused on the most vulnerable, a relatively small group of the population, which can be attended in medicalized nursing homes rather than in hospitals. Thus, reducing infections among that small group had a minor impact in both cases and hospitalizations. Second, another possible explanation is that vaccination reduced the severity of cases, hindering case detection. Indeed, since vaccination reduces the chance of suffering severe disease, it is possible that those infections might have been undetected, not contributing to lower the value of *p*_*H*_. Third, the vaccines’ effects might have been hidden by the presence of a more virulent variant of SARS-CoV-2, yielding a scenario similar to the one in late 2020. Lastly, it has been proposed that the profile of hospitalized cases shifted towards un-vaccinated groups, compensating the effect of vaccination and leaving *p*_*H*_ unaltered. Surveillance data shows that, indeed, the fraction of hospitalizations corresponding to the oldest age groups (older than 80 y.o.) steadily declined since vaccination started. Conversely, the hospitalization rate within the 40 to 49 and 50 to 59 y.o. groups increased (see Fig. S4), especially in May. Note that the vaccination of these groups started precisely in May, which explains the observed pattern. Recently, daily incidence data disaggregated by age has been made publicly available by the Ministry. Further studies should use this data to better estimate the hospitalization dynamics by age-group and its relationship with vaccination.

### Bed occupancy in Aragon

We observe that, albeit the prediction of new cases is relatively simplistic, the forecast of occupancy is remarkably good, especially during the first days of the prediction. In particular, the procedure can correctly estimate the maximum bed occupancy and the date when the tendency shifts downwards. Admittedly, the ±5% scenarios may not be suitable for other regions or other circumstances, as they were specifically tailored for the situation in Aragon (see the section Surveillance data in Aragon in the Supplementary Material). Expert opinion and local knowledge have been commonly used in the unfolding of the pandemic in the absence of data [18, 27, 29, 48]. However, their power is limited, and models should be updated as soon as new data is available [52, 53]. Fortunately, our model can be applied to the output of any incidence prediction algorithm, and thus it can be easily adapted to other regions, or more complex scenarios.

### Regional comparison of new hospital admissions

As seen in Fig. S5 and Table S2, the estimation of new hospital admissions is slightly worse in a handful of regions. A lower quality of the fit usually signals that the region changed its testing capacities, rather than other external factors such as emergence of new variants. For instance, in the Principality of Asturias, we observe that after mid-November the model tends to overestimate new admissions. The reason for this discrepancy can be that either some interventions reduced the value of *p*_*H*_, or that the surveillance system was improved, and more cases were detected. It turns out that Asturias was one of the three regions, together with the Canary Islands and the Balearic Islands, which did not use antigen tests in their surveillance system. This situation changed on November 10, 2020, which would explain why our predictions overestimate occupancy after mid-November 2020. An analogous situation can be observed from mid-January 2021 in the Balearic Islands, which can be related to a massive testing campaign using antigen tests that started on mid-January 2021 and extended up to February 2021. Lastly, we also observe a slight overestimation of occupation in the region of Madrid. In this region, the health department of the autonomous community redefined contact tracing on September 28, 2020, excluding social encounters from the surveillance system. This change was reverted on November 20, 2020. The first change would force *p*_*H*_ to increase, while the latter would produce a larger number of detected cases. The combination of both would result in a larger estimation of bed occupancy from late November, therefore nicely explaining the divergence obtained in the model.

Focusing on the estimated values for the admission delay and admission probability (Fig. 4), the regions with the most different values are those for which the prediction is worst, as previously describe. It is interesting to mention that Catalonia (CT) has the lowest admission probability, which could indicate that the region is detecting more cases than the rest. However, as shown in Fig. S6, this is not indeed the case as the observed difference is a consequence of the data alone. Certainly, there are significant differences between the values reported by the Spanish Health Ministry and the regional government of Catalonia. Using the values provided by the latter, the hospitalization probability is 8.5%, in line with the rest of the regions.

Finally, it would also be possible to hypothesize that the observed differences among the regions in which the prediction was successful to the characteristics of their populations. For instance, one of the drivers of these differences could be that some regions have a larger fraction of the population within the eldest age-groups, which are more prone to need hospital care. However, in Fig. S7, we show that the correlation between these variables is very weak and cannot explain the difference.

### Strengths and limitations

The methodology presented here, as we have shown, can easily be used evaluate the effect of interventions on the hospitalization dynamics during a pandemic. Many models found in the literature usually fit the incidence to agnostic growth models, with parameters that do not have a clear interpretation in terms of epidemic spreading, and even assume that there are no interventions during a wave. The over-parametrization of these growth models may even lead to convergence problems [3]. At the same time, the model we proposed to translate cases into occupancy is independent from the one used to estimate incidence. Hence, it is possible to substitute the latter by any other model, including complex compartmental models with advanced features such as contact tracing schemes, seasonality or multi-scale interactions.

Another important strength of the model is the use of Bayesian inference, as it allows us to integrate prior information with data in a flexible way. Besides, it just requires aggregated statistics which are relatively easy to obtain (new cases, new hospitalizations and current occupancy) even at the regional level. This way, and thanks to the interpretability of its parameters, it provides a unifying framework to study the hospitalization dynamics of multiple areas, enabling the evaluation of the effect of different policies. As we have discussed, the model can be used to detect changes in the dynamics, and to study several waves at the same time, something that models based on growth equations fitted to a single wave cannot do.

Our model also has several limitations. The use of aggregated data facilitates its applicability, but also limits its potential if individual data is available. For instance, models based on queues could use individual information to predict precisely when a specific person will be discharged. Our choice to model the incidence was heavily based on the observations in the area of interest, and might not be directly applicable in others. Note, however, that it is completely decoupled from the model of the hospital dynamics, and thus it can be improved without changing the main model. Another limitation is that to train the model it is necessary to have information of a period of a few weeks. Even though the predictions can then be used for periods much larger than the one used for training, as shown in the paper, it is not suited for the very first days of an emerging pandemic. Lastly, in Bayesian frameworks the choice of priors can be problematic, but we showed that in this case with uninformative priors convergence is correctly achieved.

## Conclusions

This analysis shows that the situation is far from being static. During the course of the epidemic in Aragon, we have identified nontrivial changes in the LoS, and noticeable modifications on the detection capabilities in other regions of Spain. Furthermore, we have seen that there are significant differences in the hospital dynamics of each region, and the quality and quantity of data provided by their institutions. Data for the same region, extracted from two different sources, can yield quite different values, which might cast doubts on its reliability. Lastly, the lack of disaggregated data on very basic characteristics, such as age, limits the conclusions that can be obtained, such as the possible role of emerging variants. This problem is especially important for underrepresented populations, for which this type of analysis cannot be performed, and who are usually those who would benefit the most from research that could unravel, induced or endemic, inequalities of the population.

Although in the absence of information the parameters that we have estimated can be used to conduct several analyses in other parts of the world, including parameterizing epidemiological models, we have also seen that there can be significant differences across regions of a relatively small country. Thus, it is important to bear this in mind when comparing the effect of the pandemic in very different regions of the world, with diverse cultures and varying resource accessibility. The explosion on the availability of almost real-time data all over the world, represents a huge opportunity for the advancement of science and the education of society. Yet, at the same time, it shows that many of our information systems are still immature and that there is much work left to be done before we are ready for future pandemics.

## Supplementary Information

**Additional file 1: Fig. S1.** Prior and posterior distributions. **Fig. S2.** Time-interval distributions. **Table S1.** Estimated parameters for the region of Aragon. **Fig. S3.** Estimated reproduction number as a function of time, R(𝑡), in Aragon. **Fig. S4.** Age profile of hospital admissions. **Table S2.** Forecast quality in each autonomous region. **Fig. S5.** Number of daily admissions to hospital in each autonomous region. **Fig. S6.** Comparison between the regional and national data for Catalonia. **Fig. S7.** Effect of the regional age distribution on the admission probability. **Fig. S8.** Robustness of the time-interval distributions. **Fig. S9.** Number of daily admissions to hospitals in the region of Aragon using an Exponential distribution for the admission delay. **Fig. S10.** Daily number of beds occupied by COVID-19 patients in Aragon using an Exponential distribution for the admission delay and a Gamma distribution for the LoS.

## Data Availability

All the data and code necessary to reproduce the results are available in https://github.com/aaleta/COVID_hospitalization and DOI 10.5281/zenodo.7558751. Due to confidentiality, the detailed data on the characteristics of hospitalized patients in Aragon cannot be shared, but all the analysis and the model estimation was conducted using publicly available data which can be accessed from the original sources, or in the GitHub folder. The former dataset was only used to estimate the shape of the prior distributions. The Supplementary Material includes robustness tests performed with other distributions unrelated to said data.

## Abbreviations

LoS: length of stay
MCMC: Markov-Chain Monte-Carlo
IQR: inter-quartile range
