# Supplementary material for: Unraveling the COVID-19 hospitalization dynamics in Spain using Bayesian inference
Source: BMC Med Res Methodol. 2023 Jan 25;23:24. doi: 10.1186/s12874-023-01842-7 (PMC9875773; doi:10.1186/s12874-023-01842-7)
Supplement: Supplementary file 1 — Additional file 1: Fig. S1. Prior and posterior distributions. Fig. S2. Time-interval distributions. Table S1. Estimated parameters for the region of Aragon. Fig. S3. Estimated reproduction number as a function of time, R(𝑡), in Aragon. Fig. S4. Age profile of hospital admissions. Table S2. Forecast quality in each autonomous region. Fig. S5. Number of daily admissions to hospital in each autonomous region. Fig. S6. Comparison between the regional and national data for Catalonia. Fig. S7. Effect of the regional age distribution on the admission probability. Fig. S8. Robustness of the time-interval distributions. Fig. S9. Number of daily admissions to hospitals in the region of Aragon using an Exponential distribution for the admission delay. Fig. S10. Daily number of beds occupied by COVID-19 patients in Aragon using an Exponential distribution for the admission delay and a Gamma distribution for the LoS. [file 12874_2023_1842_MOESM1_ESM.pdf]

Supporting Information: Unraveling the COVID-19 hospitalization dynamics in Spain using Bayesian inference

Alberto Aleta, PhD[1,4], Juan Luis Blas-Laína, M.D[2], Gabriel Tirado Anglés, M.D[3], and Yamir Moreno, Prof.[4,5,6,7]

1 ISI Foundation, Via Chisola 5, Torino 10126, Italy

2 Servicio de Cirugía General y Aparato Digestivo (Jefe de Servicio), Hospital Royo Villanova, Av San Gregorio s/n, 50015, Zaragoza, Spain

3 Unidad de Cuidados Intensivos (Jefe de Servicio), Hospital Royo Villanova, Av San Gregorio s/n, 50015, Zaragoza, Spain

4 Institute for Biocomputation and Physics of Complex Systems (BIFI), University of Zaragoza, Zaragoza 50018, Spain

5 Department of Theoretical Physics, University of Zaragoza, Zaragoza, Spain

6 Centai Institute, Torino 10138, Italy

7 Complexity Science Hub, Vienna 1080, Austria

Contents

Estimation of the model parameters.......... 2

Surveillance data in Aragon.......... 3

Regional results.......... 5

Robustness of the results.......... 8

Estimation of the model parameters

The prior and posterior distributions of the parameters for the region of Aragon are shown in figure S1. In table S1, we report the specific values obtained in the fitting, and figure S2 the corresponding distributions for the admission interval and the length of stay (LoS).

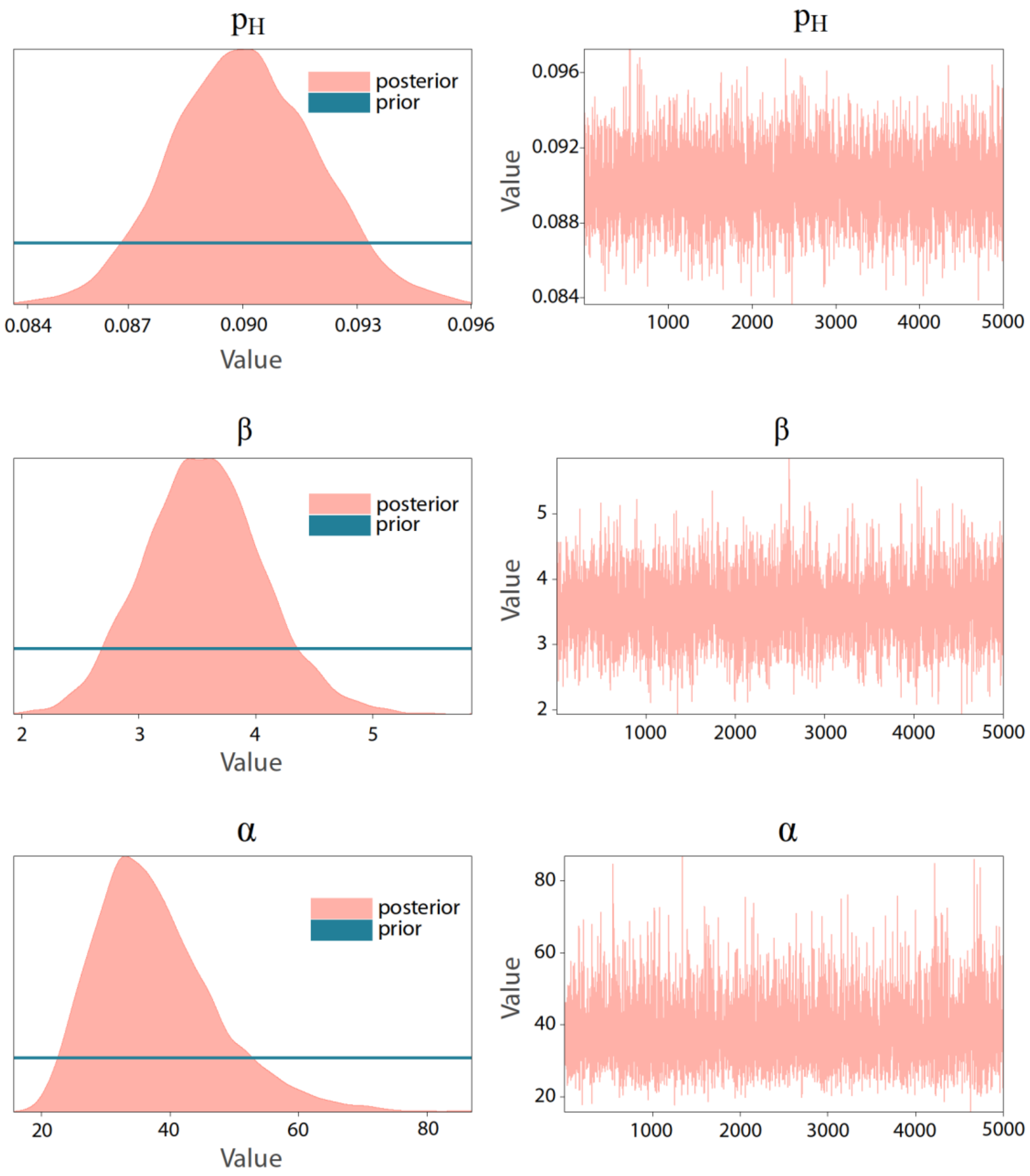

Figure S1: Prior and posterior distributions. Left column: prior and posterior distributions of the model parameters for new admissions in the region of Aragon. Right column: MCMC iterations. In all cases, the parameters were estimated for the region of Aragon using data from July 1 to December 1 2020 and their associated $\hat{R}$ was 1.0.

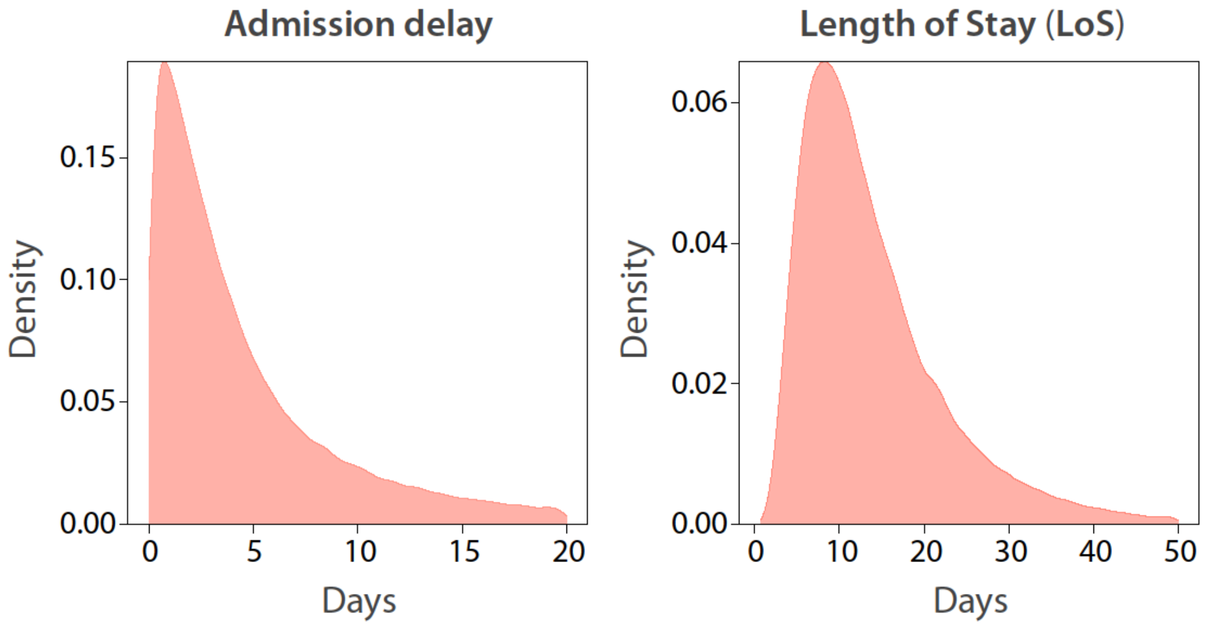

Figure S2: Time-interval distributions. Left: delay between symptom onset and hospital admission. Right: distribution of the Length of Stay (LoS) in the hospital.

Parameter	Mean	HDI 2.5%	HDI 97.5%	$\hat{R}$
p_H^{AR}	0.090	0.086	0.094	1.0
β^{AR}	3.557	2.579	4.564	1.0
μ^{AR}	2.476	2.441	2.512	1.0
σ^{AR}	0.620	0.554	0.688	1.0

Table S1: Estimated parameters for the region of Aragon. The probability of being admitted into the hospital upon being detected is p_H. The distribution governing the delay between case detection and admission is a Half-Cauchy distribution parameterized with β. The distribution on the length of stay (LoS) is modeled using a Log-Normal distribution parameterized with an average of μ and standard deviation σ.

Surveillance data in Aragon

In figure S3, we show the evolution of the reproduction number from July 1 2020 to December 1 2020 obtained from the incidence data provided by the regional government. In figure S4, we depict the age profile of the individuals admitted each week into any hospital in Aragon since vaccination started.

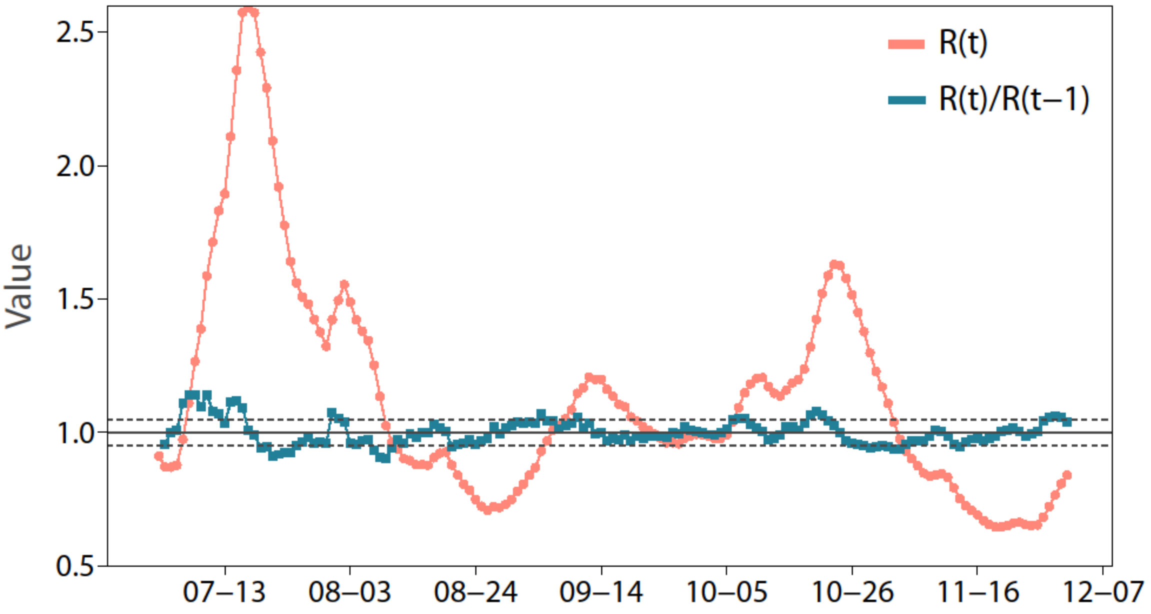

Figure S3: Estimated reproduction number as a function of time, $R(t)$, in Aragon. Circles represent the value of the reproduction number while squares show the quotient between the value at time t over the value on the previous day, $R(t)/R(t-1)$. Dashed, horizontal lines represent the values of 0.95 and 1.05.

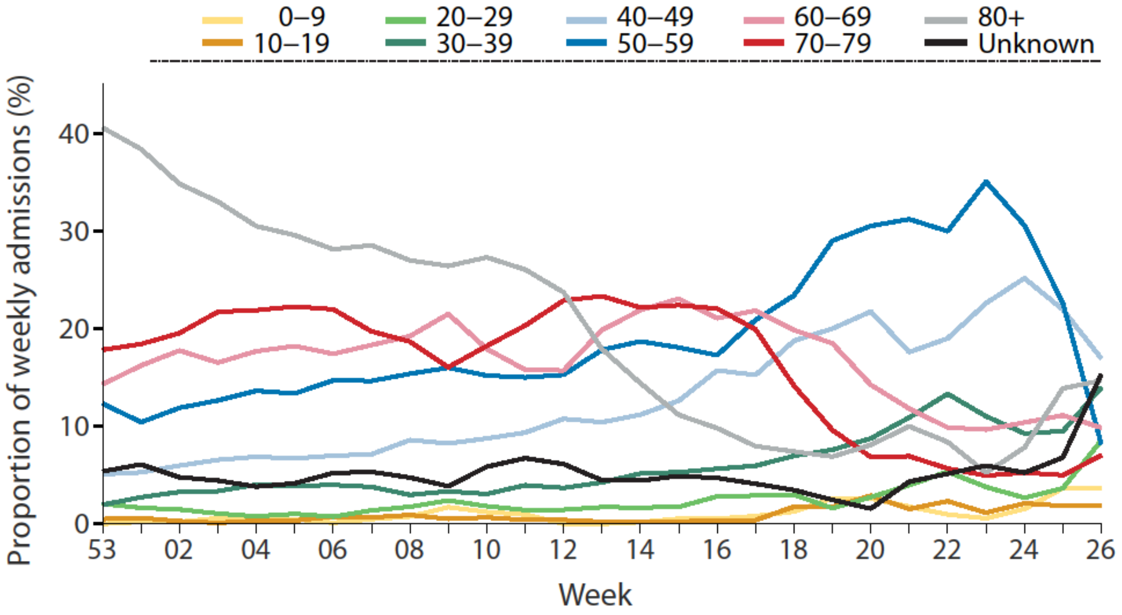

Figure S4: Age profile of hospital admissions. Proportion of weekly admissions corresponding to each age group in Aragon since vaccination started (week 53 of 2020). The curves have been smoothed using a rolling average of size 3.

Regional results

In figure S5, we show the number of new hospital admissions in each autonomous region in Spain, together with the prediction of the model. The mean absolute percentage error (MAPE) of the prediction for the third wave is presented in table S2. MAPE is defined as:

$$MAPE = \frac{100\%}{n} \sum_{t=1}^{n} \left| \frac{A_t - F_t}{A_t} \right|,$$

where A_t and F_t are the actual and forecasted values at time t, respectively.

Autonomous Region	ISO Code	MAPE (%)			
		January	February	March	April
Andalusia	AN	6.94	8.87	7.58	6.65
Aragon	AR	5.60	5.27	5.09	4.71
Principality of Asturias	AS	59.54	61.59	53.47	49.69
Canary Islands	CN	7.41	7.00	7.51	7.51
Cantabria	CB	18.98	18.18	19.43	20.47
Castille-La Mancha	CM	10.48	7.27	5.63	4.37
Castille and Leόn	CL	5.01	4.53	3.93	4.09
Catalonia	CT	4.79	5.36	4.61	4.08
Extremadura	EX	36.65	35.93	31.29	29.17
Galicia	GA	2.47	2.63	2.37	2.37
Balearic Islands	IB	15.43	10.15	10.59	12.60
La Rioja	RI	19.50	17.46	14.77	13.50
Community of Madrid	MD	20.13	30.38	32.27	32.02
Region of Murcia	MC	13.84	19.62	17.13	15.44
Chartered Community of Navarre	NC	9.92	7.94	6.96	6.22
Basque Country	PV	7.30	6.34	7.40	8.12
Valencian Community	VC	8.97	12.72	13.88	15.20

Table S2: Forecast quality in each autonomous region. For each region, the MAPE is estimated using the cumulative number of admissions up to the month represented in the table. In regions with high MAPE might have undergone changes in their contact tracing and testing procedures either during the training process or during the prediction phase.

In figure S6, we compare the data on cases and hospitalizations in Catalonia provided by the regional government and the central government. Lastly, in figure S7, we test the hypothesis that the differences in p_H across regions could be related to the fraction of their population over 64 y.o. Note that there is a clear outlier, the Principality of Asturias (AS). If we remove it, the Pearson correlation is 0.23 with p-value = 0.39, rejecting the correlation. Similarly, the Spearman correlation yields 0.43 with p-value = 0.085 if we include AS, and 0.32 with p-value = 0.23 if we exclude it, once again rejecting the hypothesis. These correlations were computed using R 4.2.1.

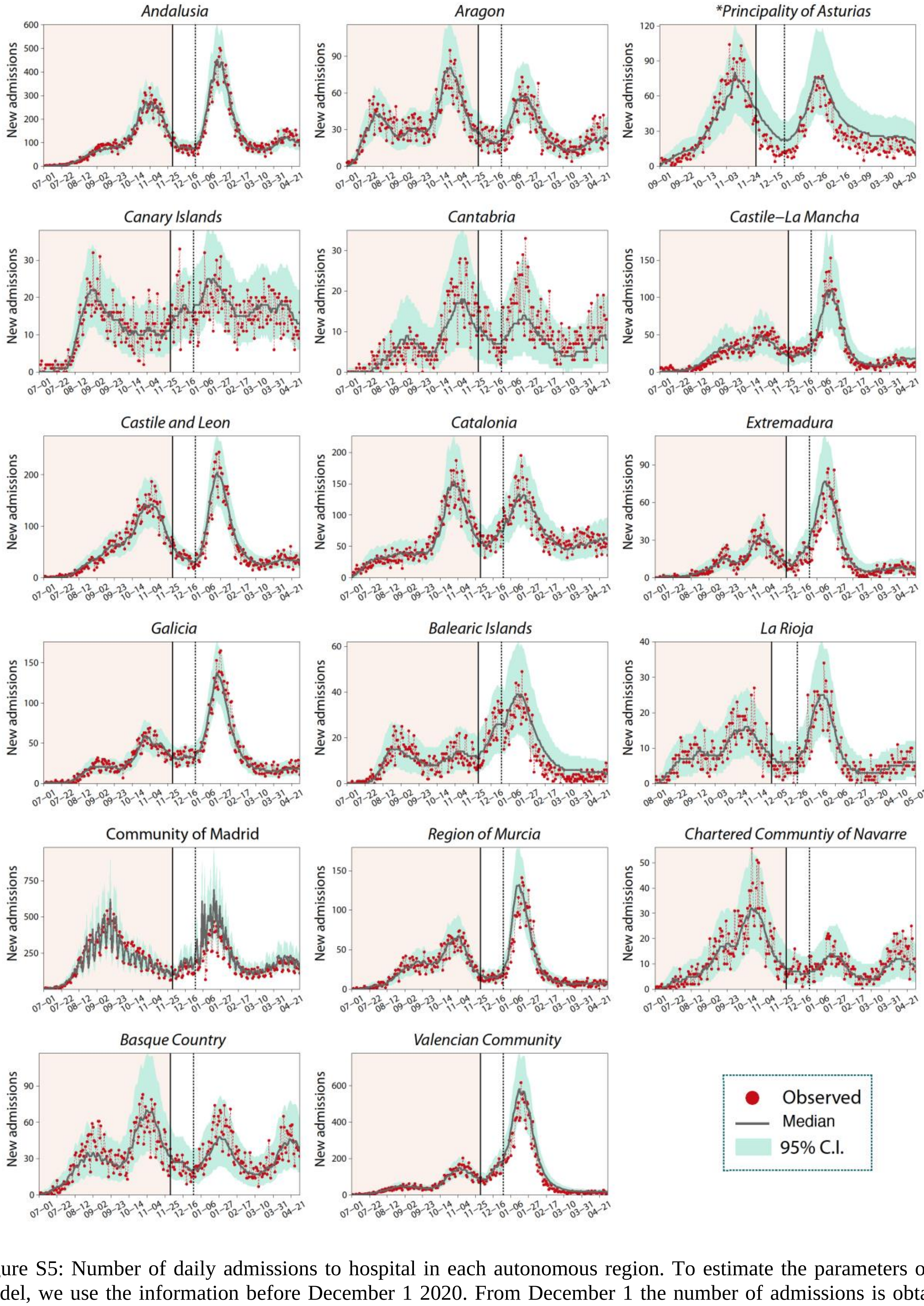

Figure S5: Number of daily admissions to hospital in each autonomous region. To estimate the parameters of the model, we use the information before December 1 2020. From December 1 the number of admissions is obtained using the estimated parameters for each region and the daily incidence. The solid line indicates the end of the training period and the dashed line the beginning of vaccine roll-out.

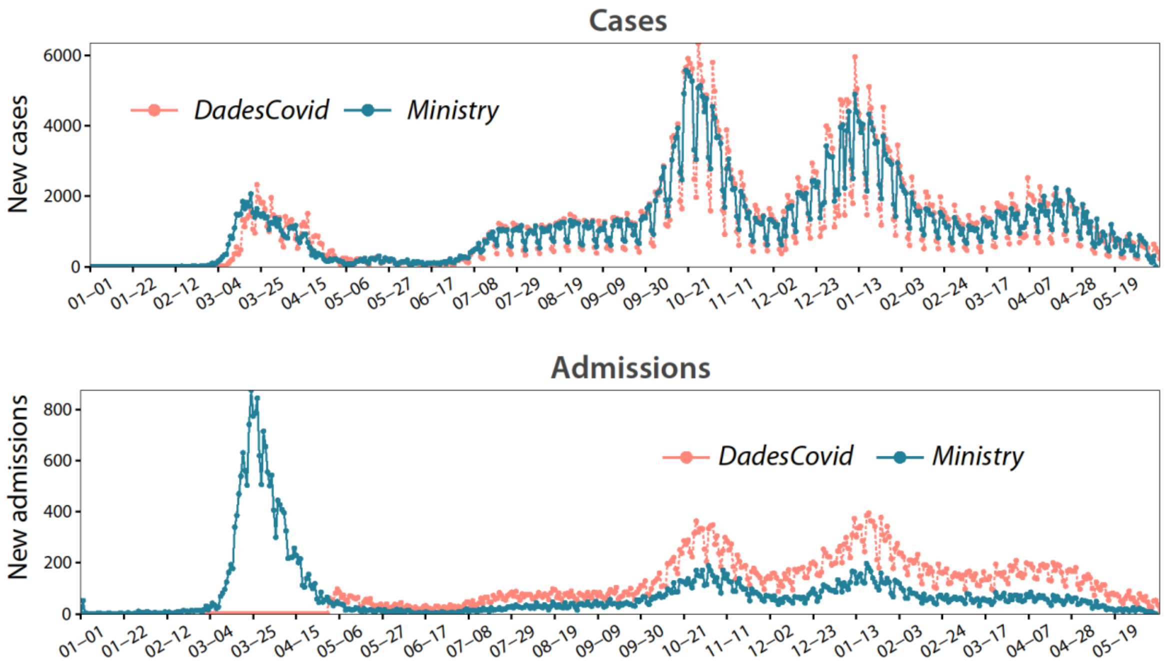

Figure S6: Comparison between the regional and national data for Catalonia. Top: number of new cases reported by DadesCovid (open data portal of the regional government) and by the Spanish Ministry of health. Bottom: number of new hospital admissions. Even though the number of new cases reported by both institutions agree, the number of new hospitalizations reported by the regional government is twice as large as the one reported by the ministry.

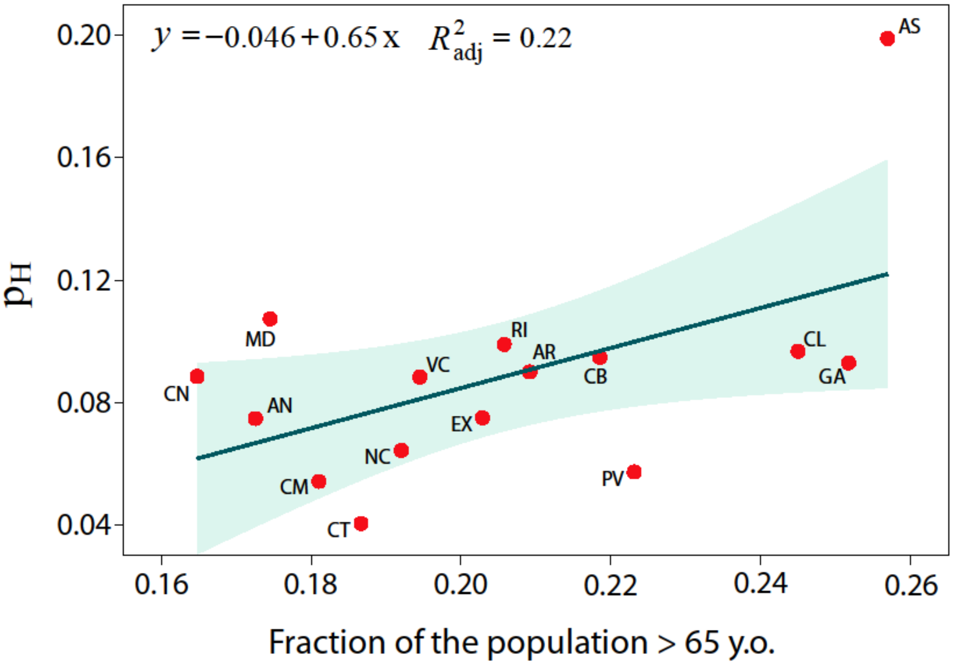

Figure S7: Effect of the regional age distribution on the admission probability. Correlation between the estimated value of p_H in each region and the fraction of the population older than 65 years old. We obtain $R^2 = 0.22$ signaling that the regression is not able to explain these differences. Furthermore, if we remove the data from the Principality of Asturias (AS) the correlation disappears completely.

Robustness of the results

We selected the Half-Cauchy and Log-Normal distributions used in the model based on the AIC value extracted from fitting several distributions to the data of Aragón. However, the differences were small and there is no underlying mechanistic reason that would make us choose one distribution over the others. As such, in this section we test the robustness of our method, repeating the analysis with other distributions for the admission and discharge processes.

In particular, we have changed the Half-Cauchy distribution to an Exponential distribution:

$$f(x|\lambda) = \lambda \exp\{-\lambda x\}, \qquad (S1)$$

where λ is the rate parameter. Similarly, we tested a Gamma distribution for the LoS:

$$f(x|\alpha,\beta) = \frac{\beta^{\alpha} x^{\alpha-1} e^{-\beta x}}{\Gamma(\alpha)}, \qquad (S1)$$

where α is the shape parameter and β is the rate parameter.

In figure S8, we show the distributions obtained with this method, compared to the ones used in the main text (figure S2). The vertical dashed lines represent the median value of the distribution. We observe some differences in the shape of the distributions, as expected given their different functional form. Nonetheless, the median value is very similar in both cases, signaling that the model is robust to this choice and tends to accommodate the proposed distributions to the hidden underlying dynamics.

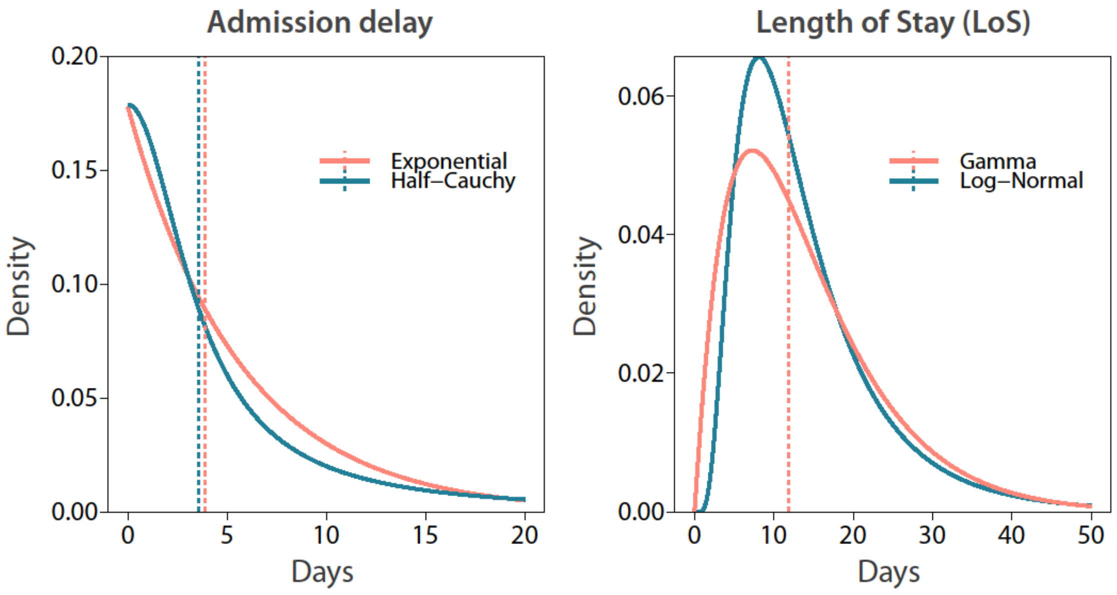

Figure S8: Robustness of the time-interval distributions. Left: delay between symptom onset and hospital admission. The Half-Cauchy distribution used in the main text is compared with an Exponential distribution fitted to the data (rate parameter 0.178 [0.143-0.217]). Right: distribution of the Length of Stay (LoS) in the hospital. The Log-Normal distribution used in the main text is compared with a Gamma distribution fitted to the data (shape parameter: 2.058 [1.659-2.486]; rate parameter: 0.145 [0.116-0.176]). In both plots the vertical, dashed line indicates the median value of the distribution. Note that the median values for the LoS are completely overlapped.

Figures S9 and S10 show the estimated admissions and bed occupancy, respectively, using the new distributions (Exponential for the admissions and Gamma for the LoS). We observe that the model can estimate adequately both quantities, with results equivalent to the ones presented in the main text. The estimated admission probability is 0.087 [0.083 – 0.090] while in the original model it was 0.090 [0.086-0.094].

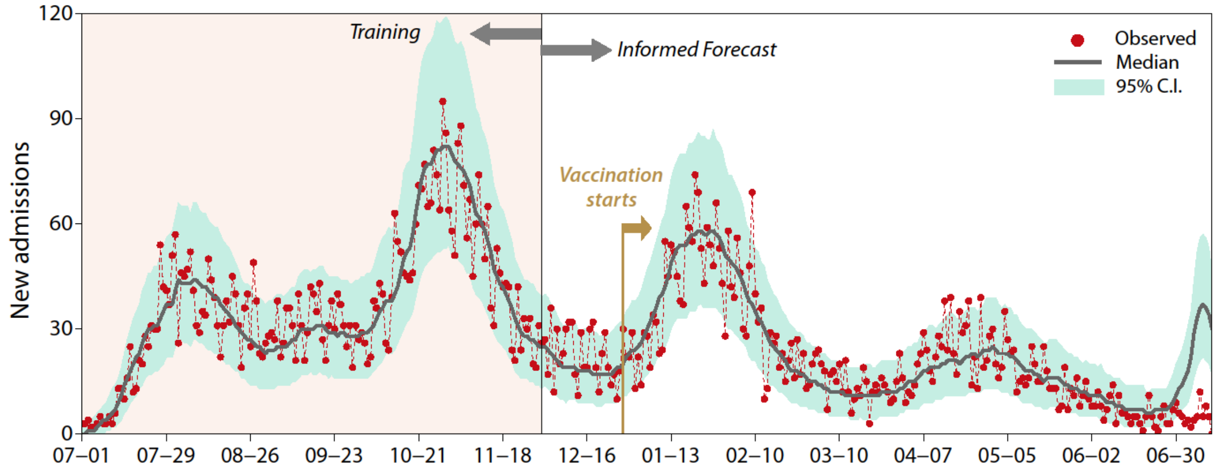

Figure S9: Number of daily admissions to hospitals in the region of Aragon using an Exponential distribution for the admission delay. The estimated admission probability upon detection is p_H = 0.087 [0.083-0.090].

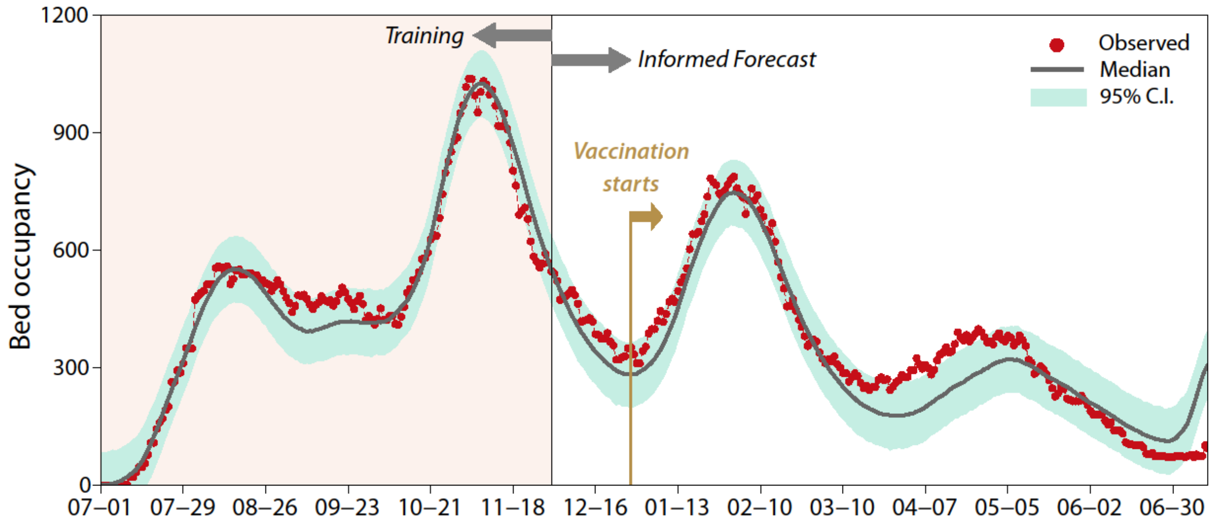

Figure S10: Daily number of beds occupied by COVID-19 patients in Aragon using an Exponential distribution for the admission delay and a Gamma distribution for the LoS.
